# geno2pheno[ngs-freq]: a genotypic interpretation system for identifying viral drug resistance using next-generation sequencing data

**DOI:** 10.1093/nar/gky349

**Published:** 2018-05-01

**Authors:** Matthias Döring, Joachim Büch, Georg Friedrich, Alejandro Pironti, Prabhav Kalaghatgi, Elena Knops, Eva Heger, Martin Obermeier, Martin Däumer, Alexander Thielen, Rolf Kaiser, Thomas Lengauer, Nico Pfeifer

**Affiliations:** 1 Department of Computational Biology and Applied Algorithmics, Max Planck Institute for Informatics, Saarland Informatics Campus, 66123 Saarbrücken, Germany; 2 Institute of Virology, University of Cologne, Fürst-Pückler-Str. 56, 50935 Cologne, Germany; 3 MVZ Medizinisches Infektiologiezentrum Berlin (MIB), Oudenarder Str. 16, 13353 Berlin, Germany; 4 Seq-IT, Pfaffpl. 10, 67655 Kaiserslautern, Germany; 5 Methods in Medical Informatics, Department of Computer Science, University of Tübingen, Sand 14, 72076 Tübingen, Germany; 6 Medical Faculty, University of Tübingen, Geissweg 5, 72076 Tübingen, Germany

## Abstract

Identifying resistance to antiretroviral drugs is crucial for ensuring the successful treatment of patients infected with viruses such as human immunodeficiency virus (HIV) or hepatitis C virus (HCV). In contrast to Sanger sequencing, next-generation sequencing (NGS) can detect resistance mutations in minority populations. Thus, genotypic resistance testing based on NGS data can offer novel, treatment-relevant insights. Since existing web services for analyzing resistance in NGS samples are subject to long processing times and follow strictly rules-based approaches, we developed geno2pheno[ngs-freq], a web service for rapidly identifying drug resistance in HIV-1 and HCV samples. By relying on frequency files that provide the read counts of nucleotides or codons along a viral genome, the time-intensive step of processing raw NGS data is eliminated. Once a frequency file has been uploaded, consensus sequences are generated for a set of user-defined prevalence cutoffs, such that the constructed sequences contain only those nucleotides whose codon prevalence exceeds a given cutoff. After locally aligning the sequences to a set of references, resistance is predicted using the well-established approaches of geno2pheno[resistance] and geno2pheno[hcv]. geno2pheno[ngs-freq] can assist clinical decision making by enabling users to explore resistance in viral populations with different abundances and is freely available at http://ngs.geno2pheno.org.

## INTRODUCTION

Drug resistance mutations can emerge rapidly in patients infected with pathogens such as human immunodeficiency virus type 1 (HIV-1) or hepatitis C virus (HCV). Since viral resistance can severely impact the success of antiretroviral therapy, genotypic resistance testing is performed when treatment is initiated or in case of treatment failure. Genotypic resistance tests consist of two steps: sequencing the relevant segments of the viral genome followed by the interpretation of drug resistance based on the amino-acid sequence (1). There exist two approaches for interpreting drug resistance: rules-based interpretation systems and algorithm-driven interpretation systems. While rules-based interpretation systems rely on the knowledge of expert panels, algorithm-driven systems are based on statistical models that are trained on clinical or virological data using machine learning algorithms. The spectrum of expert opinions has given rise to several sets of rules, for example, the rule sets from ANRS, HIVdb (2), HIV-GRADE and the Rega institute, all of which are available via the HIV-GRADE website (3). Similarly, algorithm-driven approaches differ among each other with respect to the applied machine learning algorithms and the data sets that are used for training the models. For example, geno2pheno[resistance] (4–7) uses support vector regression and classification, while the more recent SHIVA software (8) employs random forests.

Despite their differences, all existing genotypic resistance interpretation systems share one commonality: They interpret data from Sanger sequencing, a technology that has dominated the field due to its cost effectiveness and low rate of errors. However, with a detection limit of 10%-20% (9,10), Sanger sequencing is unable to identify resistance mutations in minority populations. Next-generation sequencing (NGS), on the other hand, allows for the identification of variants even at low abundances (11,12). Due to the potential clinical relevance of minority resistance mutations (13,14) and the decreasing costs of NGS, the implementation of NGS for viral resistance analysis has increased considerably in recent years. Still, few web services for interpreting NGS data with respect to drug resistance are available. To our best knowledge, the only existing web services for this purpose are PASeq and HyDRA (15). These tools require the raw sequencing data resulting from subjecting an HIV-1 sample to NGS (e.g. as a FASTQ, gzipped FASTQ, or SFF file). After a sample has been uploaded, a processing pipeline performs the following tasks: (i) reads are trimmed in order to remove low-quality positions; (ii) reads are mapped to a reference sequence; (iii) the abundance of mutations is quantified and (iv) resistance is inferred. In contrast to web services for interpreting Sanger sequences, which provide results immediately, PASeq and HyDRA perform more time-intensive computations and notify users via email when the results become available. Both PASeq and HyDRA support only rules-based interpretations and use Stanford's popular HIVdb by default, although HyDRA also allows for the consideration of user-defined sets of rules.

In this work, we present geno2pheno[ngs-freq], a web service for identifying resistance in NGS samples of HIV-1 and HCV that is based on the well-established methods of geno2pheno[resistance] (4–7) and geno2pheno[hcv] (16). geno2pheno[ngs-freq] does not require the input of raw sequencing data and instead relies on frequency files that tabulate either the counts of observed nucleotides or codons along a viral genome. In contrast to raw NGS data, whose sheer size may prevent some labs from performing resistance analyses over the internet, frequency files are quite small. Since geno2pheno[ngs-freq] does not need to map thousands of reads to a reference sequence, batches of frequency files can be analyzed quickly and results can be inspected immediately.

## MATERIALS AND METHODS

In the following sections, we introduce the frequency file format, illustrate the mechanisms behind geno2pheno[ngs-freq], and outline how we validated the web service.

### Format of input files

**Figure 1.**
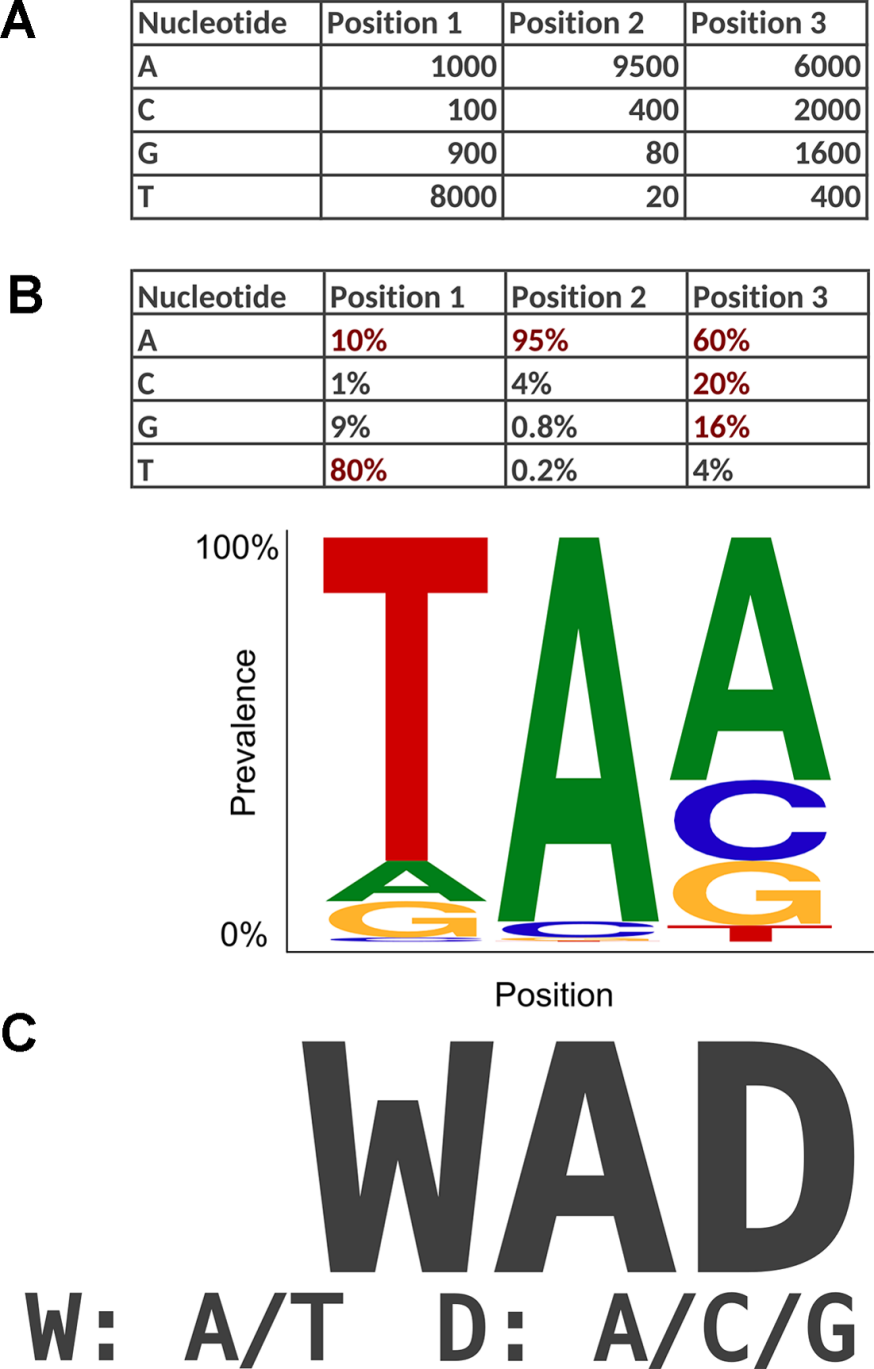
Transformation of a single-nucleotide frequency file to a consensus sequence at a prevalence cutoff of 10%. (**A**) Example of a nucleotide frequency file providing the nucleotide counts for the first three positions in a viral genome. (**B**) Table of prevalence ratios in which observations with ratios of at least 10% are shown in red. The corresponding motif logo in which the height of individual nucleotides reflects their prevalence is shown below. (**C**) Consensus sequence constructed for a prevalence cutoff of 10%. Ambiguous positions are encoded according to IUPAC nomenclature.

Frequency files are CSV files containing either the counts of observed codons (Supplementary File S1) or nucleotides (Figure 1A and Supplementary File S2) along a viral genome. These files can be generated via custom or available NGS processing pipelines such as VirVarSeq (17) or MinVar (18). In the following, we consider a frequency file as a matrix $F \in \mathbb{N}_0^{m \times n}$ whose number of rows $m \in \mathbb{N}$ is determined by the number of genomic positions and whose number of columns $n \in \mathbb{N}$ is defined either by the number of nucleotides or triplets. Let $\mathcal{A}\ = \{ - ,\ A,\ C,\ T,\ G,\ N\}$ be the nucleotide alphabet and let ${\mathcal{A}_3} = {(\mathcal{A}\backslash \{ - ,\ N\} )^3}\ \ \cup \{ ( - , - , - )\}$ be the triplet alphabet. Nucleotide frequency files contain entries ${f_{ij}}$ that denote the number of reads supporting the nucleotide $j \in \mathcal{A}$ at position $i$, while codon frequency files are defined by entries ${f_{ij}}$ where $j \in {\mathcal{A}_3}$ relates to triplets instead.

### Workflow

Once a user has supplied a set of prevalence cutoffs, a frequency file, and an optional sample identifier, geno2pheno[ngs-freq] performs the following steps: (i) generation of a consensus sequence for every prevalence cutoff; (ii) inference of the viral species and (iii) identification of viral resistance for every consensus sequence. Once all computations have completed, users can contrast the estimated impact of identified variants at low abundances with those at greater abundances by selecting two cutoffs for displaying the results, the *personal* and the *reference cutoff*. By default, the personal cutoff is set to 2%. The default setting should ensure tolerance with regard to sequencing errors for all NGS platforms suitable for viral samples (19,20), and, at the same time, allow for the consideration of clinically relevant minority variants (21). The default reference cutoff for samples from HIV-1 is set to 10% (22) and to 15% for samples from HCV (23) such that results based on the reference cutoff should agree well with those that would be obtained via Sanger sequencing.

#### Generation of consensus sequences

For each prevalence cutoff ${c_F} \in [ {0,1} ]$ provided for a frequency file $F \in \mathbb{N}_0^{m \times n}$, the web service generates a consensus sequence in the following manner. Let ${d_i} = \ \mathop \sum\nolimits_j {f_{ij}}$ indicate the depth of coverage at position $i \in \{ {1,\ \ldots ,\ m} \}$ in the frequency file. If $F$ is a codon frequency file, the ratio at which the codon $j \in {\mathcal{A}_3}$ occurs at position $i$ is determined by ${x_{ij}} = \frac{{{f_{ij}}}}{{{d_i}}}$, and, if $F$ is a single-nucleotide file, as described in Supplementary Text S3. We construct the consensus sequence ${s_{{c_F}}}$ by considering only ${\mathcal{A}_{i,{c_F}}} = {\rm{\{ }}j{\rm{|}}{x_{ij}} \ge {c_F}\}$, the set of observations whose prevalence is at least ${c_F}$, and setting position $i$ of the consensus sequence as ${s_{i,{c_F}}} = {\rm{\Phi (}}{\mathcal{A}_{i,{c_F}}}{\rm{)}}$. In case that ${\mathcal{A}_{i,{c_F}}}$ is empty (i.e. no frequencies are greater or equal to the cutoff), we use the greedy criterion, ${\mathcal{A}_{i,{c_F}}} = {\rm{argma}}{{\rm{x}}_j}{x_{ij}}$ instead. The function $\phi$ translates nucleotides or codons into their corresponding IUPAC representation (24). Given a prevalence cutoff of ${c_F} = \ 10\%$ and the observed prevalence ratios ${x_{iA}} = \ 10\% ,{\rm{\ }}{x_{iC}} = \ 1\% ,{\rm{\ }}{x_{iG}} = \ 9\%$, and ${x_{iT}} = \ 80\%$ (Figure 1B), we would set ${s_{i,10\% }} = \ {\rm{\Phi (}}{\mathcal{A}_{i,10\% }}{\rm{)\ }} = \ {\rm{\Phi (}}A,\ T{\rm{)}} = \ W$ (Figure 1C).

In order to correctly extract the target amplicon from a frequency file, we truncate the sequence by combining a relative and an absolute cutoff. Let ${d_{{\rm{med}}}}{\rm{\ }}$ denote the median depth of coverage over all positions with non-zero coverage. We set the coverage cutoff to ${d_{{\rm{cut}}}} = \ {\rm{max}}( {20,{\rm{\ min}}( {100,\ 0.1 \cdot {d_{{\rm{med}}}}} )} )$ and use it to define the start of the target region as ${i_s} = \mathop {{\rm{min}}}\nolimits_i {\rm{\{ }}i \in \{ {1,\ \ldots ,\ m} \}\ {\rm{|}}\ {{\rm{d}}_{\rm{i}}} \ge {d_{{\rm{cut}}}}\}$. If ${i_s}$ is undefined, no further computations are performed. Otherwise, we define the end of the region as ${i_e} = \mathop {{\rm{min}}}\nolimits_i {\rm{\{ }}i \in \{ {{i_s} + 1,\ \ldots ,\ m} \}\ {\rm{|}}\ {d_i} < 0.5 \cdot {d_{{\rm{cut}}}}\} \ - 1$. If ${i_e}$ is undefined, we set ${i_e}$ to the value of $m$.

Please note that geno2pheno[ngs-freq] provides warnings for individual positions $i$ with ${d_i} < 100$, when the 25^th^ percentile of a genomic region is smaller than 100, or when stop codons or frameshift mutations are found in a genomic region. In the following, we use the term *default consensus sequence* to denote the consensus sequence of a sample that was constructed according to the corresponding default reference cutoff (i.e. ${s_{10\%}}$ for HIV-1 and ${s_{15\% }}$ for HCV samples).

#### Inference of the viral species

We identify the viral species from which an input sample originates by aligning its default consensus sequence to the genomic segments of the reference sequences for HIV-1 and HCV, HXB2 (25) and H77 (26), respectively. To ensure that we perform resistance analyses only for the supported viral species, we consider only high-quality alignments, i.e. alignments with high similarities between query and reference sequence. If no high-quality alignments are available, it is assumed that the sample derives from a species that is not supported and no further computations are performed. Otherwise, the annotated species of the reference sequence with the greatest alignment score is used. An alignment is considered a high-similarity alignment if it satisfies two similarity criteria, which are defined by dividing the number of matching amino acids in the alignment either by the length of the alignment (*alignment similarity*) or by the length of the reference sequence (*reference similarity*). For HIV-1 sequences, a minimal alignment similarity of 60% and a minimal reference similarity of 50% is used for all regions, except for the reverse transcriptase (RT). Since all major drug resistance mutations are located within the first half of the gene, the RT region is frequently merely partially amplified. Thus, we require a reference similarity of only 20% for the RT. Due to the greater phylogenetic divergence of HCV, we require an alignment similarity of 40% and a reference similarity of 20% for all HCV regions.

#### Identification of viral resistance

Viral resistance of HIV-1 and HCV samples is interpreted using the approaches of geno2pheno[resistance] (4–7) and geno2pheno[hcv] (16), respectively. geno2pheno[resistance] provides two types of approaches. The original *g2p[resistance]* approach relies on support vector regression models with linear kernel functions. These models were trained on genotype-phenotype pairs consisting of Sanger sequences from HIV-1 and corresponding measurements of drug-specific resistance factors (RF), which quantify the fold change in the half maximal inhibitory concentration of a mutated sample with respect to the wildtype (6,27). The more recently developed approach of *g2p[drug-exposure]* is based on support vector classification models. These models were trained using clinical data consisting of Sanger sequences and corresponding labels indicating whether a sequence originates from a patient that had received treatment with a specific drug (7). This approach estimates a quantity that is correlated with the degree of drug exposure, the so-called drug-exposure score (DES). Because RFs and DESs vary considerably across drugs, geno2pheno[resistance] standardizes these quantities to *z*-scores providing the number of standard deviations that a value is above/below the mean of therapy-naïve patients. Finally, each z-score is transformed to one of three interpretable, clinically-motivated levels of resistance (5): *susceptible, intermediate*, or *resistant*.

geno2pheno[hcv], on the other hand, relies on a set of drug- and genotype/subtype-specific rules that was chosen by an expert panel through extensive reviewing and weighting of literature related to HCV drug resistance. The level of drug resistance associated with an input sequence is determined by scanning the amino acids of nonstructural protein 3 (NS3), nonstructural protein 5A (NS5A), and non-structural protein 5B (NS5B) for matches to any of the rules and reporting the worst-case resistance level. For example, given a virus with subtype 1b, the mutation 41R would not affect susceptibility to the NS3 inhibitor asunaprevir, however, susceptibility would be considered to be reduced if both 41R and 80R were present. geno2pheno[ngs-freq] uses the following outcomes for classifying the resistance of HCV samples to individual drugs: *susceptible, substitution on scored position* (substitution at a position for which a rule exists), *resistance-associated mutation in closest subtype* (for rare subtypes only: existence of a rule in the closest non-rare subtype), *reduced susceptibility, resistant*, and *unlicensed* (drug is not approved for the identified subtype).

Please note that both approaches yield warnings when resistance-associated positions are missing from the constructed consensus sequences.

### Validation

For validating the web server, we analyzed a total of 3844 frequency files of which 926 files represented samples from HIV-1 (24.1%) and 2918 files represented samples from HCV (75.9%). Resistance interpretations were obtained for 922 of 926 HIV-1 samples (99.6%) and 2898 of 2918 HCV samples (99.3%). For the remaining samples, geno2pheno[ngs-freq] did not provide a result due to low depth of coverage. Since we had re-implemented the approach of geno2pheno[hcv] during the development of geno2pheno[ngs-freq], we investigated the concordance between the predictions of geno2pheno[ngs-freq] and geno2pheno[hcv] using the default consensus sequences constructed from the 2866 successfully analyzed HCV frequency files. We did not perform an analogous validation for the HIV-1 samples because predictions for HIV-1 samples are based on the current version of geno2pheno[resistance].

### Technical details

The geno2pheno backend is implemented in C++ and relies on an Oracle database for data storage. We implemented the frontend with Typescript and the React library. The web interface allows for the analysis of batches containing at most 20 files.

## RESULTS

### Validation

The levels of resistance that were predicted with geno2pheno[ngs-freq] and geno2pheno[hcv] for the default consensus sequences had an agreement of 99.7%. The median runtimes required for analyzing HIV-1 and HCV samples were 6 seconds and 4 seconds, respectively.

### Case studies

In this section, we provide two case studies that illustrate how geno2pheno[ngs-freq] can offer insights that may impact clinical decision making. While non-nucleoside reverse transcriptase inhibitor (NNRTI) resistance mutations at low abundances are associated with virological failure (14,28–30), it is still generally unclear how minority resistant variants influence the treatment outcomes of HIV-1 infected persons (31–36). The impact of HCV minority resistant variants is less studied than for HIV-1 but the presence of minority resistant variants has recently been shown to deteriorate the outcomes in subtype 1 patients being treated with NS5A inhibitors (37). Although treatment choices based on minority resistant variants should be taken with particular care as noted previously (38), regimens that could be impaired by resistant minorities can be excluded if suitable alternative treatment options are available.

The case studies can be replicated by visiting http://ngs.geno2pheno.org, ensuring that the default prevalence cutoffs (2%, 10% and 15%) are selected, and loading the frequency files that are provided through Supplementary Files S1 and S2. The HIV-1 case study was performed using the *g2p[resistance]* model, which predicts phenotypic drug resistance (27), while the HCV case study was performed using the geno2pheno[hcv] rule set (16).

#### Case study 1: HIV-1 resistance interpretation

**Figure 2.**
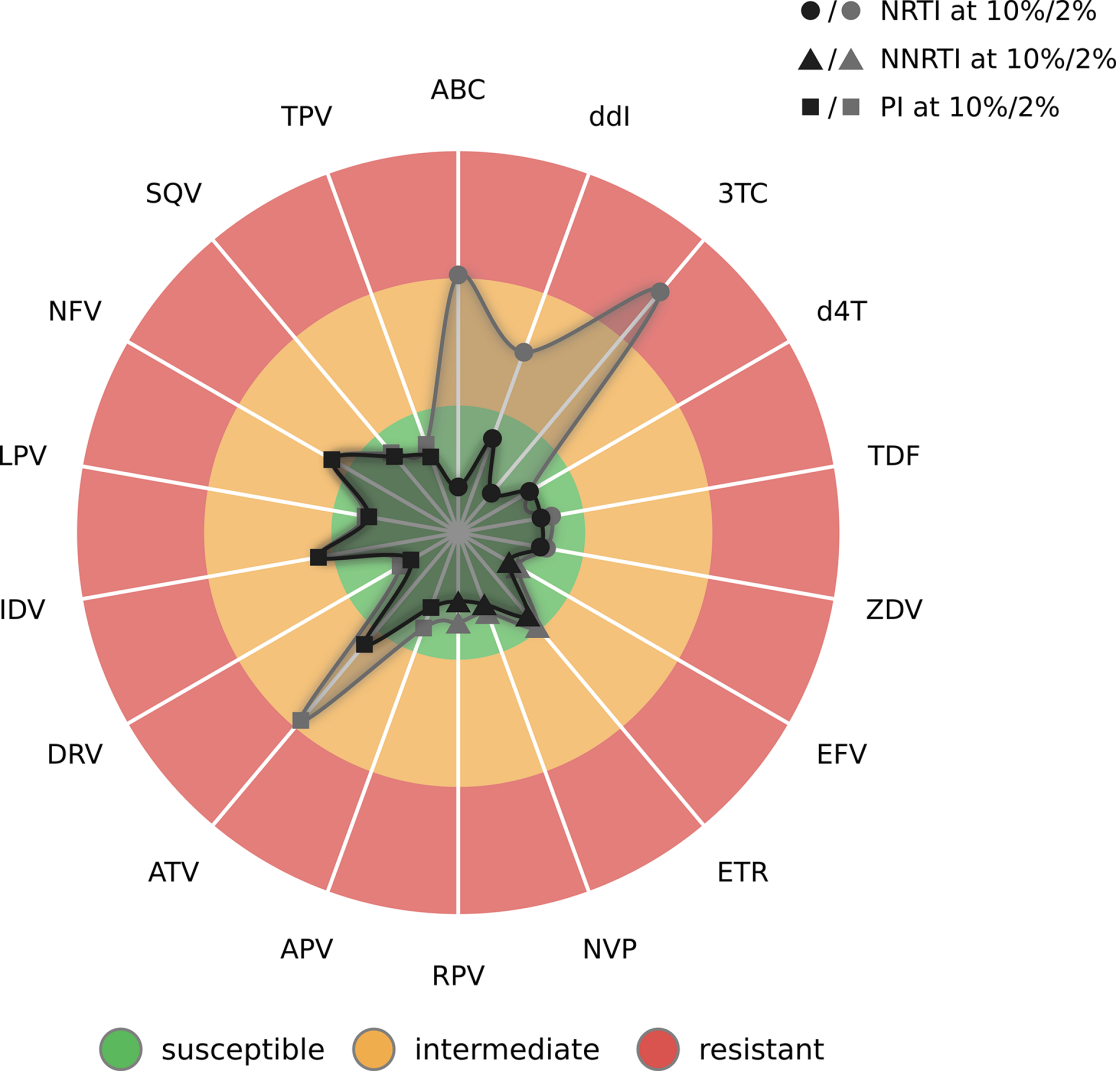
Radar plot of predicted drug resistance for the HIV-1 sample from the first case study. Each spoke in the plot relates to an antiretroviral drug. Each drug class is represented by a different type of symbol. Here, circles, triangles, and squares indicate the results for nucleoside reverse transcriptase inhibitors (NRTIs), non-nucleoside reverse transcriptase inhibitors (NNRTIs), and protease inhibitors (PIs), respectively. The three colored circle sectors indicate the estimated levels of drug resistance, from inside to outside: green for susceptibility, orange for intermediate resistance, and red for resistance. Resistance levels are indicated by two surfaces. The inner surface shows the estimated level of resistance for the consensus sequence based on the reference prevalence cutoff at 10%, while the outer surface indicates the level of resistance for the consensus sequence based on the personal cutoff at 2%. The points defining the surfaces are determined through the z-scores that are predicted by geno2pheno[resistance]. Points lying further to the inside of the plot indicate decreased drug resistance, while points lying further to the outside indicate increased resistance.

This case study (Supplementary File S1) is based on a plasma isolate from an HIV-1 infected patient with a viral load of 102 000 copies/ml. The plot of viral drug resistance (Figure 2) reveals that the major viral population (at the reference cutoff of 10%) seems to be susceptible to nearly all drugs. When considering also minor viral populations (at the personal cutoff of 2%) we find highly increased levels of resistance to ABC, ddI and 3TC, drugs from the class of nucleoside reverse transcriptase inhibitors (NRTIs). Using the resistance table, we can determine that the increased level of resistance is caused by the well-studied resistance mutation M184V, which occurs at a population prevalence of 2.36%. M184V is not only known for enhancing the susceptibility to the NRTIs ZDV, d4T and TDF, but also for delaying the emergence of resistance to these drugs (39). Therefore, a combination therapy consisting of two such NRTIs and one protease inhibitor such as TDF + ZDV + DRV would be a reasonable choice. An alternative treatment with fewer side effects could consist of TDF + FTC + DRV. The idea behind this treatment is that FTC could stabilize M184V such that susceptibility to TDF is ensured (39). Moreover, although M184V is associated with a >100-fold reduction in susceptibility to FTC *in vitro* (40), FTC exhibits residual activity in the presence of M184V *in vivo* (41,42). Therefore, even if the minority population characterized by M184V were to become the major viral population over time, FTC would still be residually active.

#### Case study 2: HCV resistance interpretation

The visualization of resistance for the provided HCV sample (Supplementary File S2) reveals an interesting scenario (Supplementary Figure S4). Although the viral population at the 15% cutoff seems to be susceptible to all direct- acting antiviral agents targeting NS5A, the population at the 2% cutoff seems to be resistant to most NS5A inhibitors due to the presence of the resistance mutation 30R, which was found at a prevalence of 6.1%. Using this information, the treating clinician may decide to avoid the use of the NS5A inhibitors DCV, EBR, LDV, and OBV, for which resistance was reported, and instead use VEL, to which the viral population seems to be susceptible.

## DISCUSSION

In contrast to existing web services for interpreting viral resistance for NGS samples, geno2pheno[ngs-freq] uses frequency files instead of raw NGS data, which offers many benefits. First, due to their small size (kilobytes vs megabytes), samples can be uploaded quickly even in settings with limited bandwidth. Second, resistance interpretation does not require the time-intensive step of processing the raw NGS data, allowing for rapid analyses (a few seconds vs several minutes or hours). Third, the use of frequency files offers greater flexibility than basing the analysis on raw sequencing data since interpretation engines based on the latter data apply pre-determined pipelines for the basic processing of the NGS samples. By relying on frequency files, geno2pheno[ngs-freq] does not impose limitations on the manner in which NGS samples are processed.

Of course, using frequency files also entails loss of information. For nucleotide frequency files, amino acid frequencies need to be estimated and spurious amino acids may be generated. Imagine that the codons ATA (*Ile*) and TTT (*Phe*) are observed at the same genomic position. In this case, the triplets ATT (*Ile*) and TTA (*Leu*) would be considered in addition to the observed nucleotides when constructing the consensus sequence. Thus, the unobserved amino acid *Leu* would be erroneously taken into account during the resistance interpretation, which may lead to an incorrect estimate of drug resistance. Therefore, we generally recommend the use of codon frequency files because this file format retains information on the abundance of tri-nucleotides. Thus, amino-acid frequencies are represented correctly and the appropriate translation of codons containing multiple ambiguous positions can be determined. Another limitation of frequency files is that they do not allow for quasispecies reconstruction. While quasispecies reconstruction may offer insights in some scenarios, the mediocre precision/recall trade-off of most methods for inferring quasispecies (43) suggests that these approaches are not yet mature enough for routine use. However, more recent approaches seem more promising (44,45). Last, future resistance interpretation systems based on read-based models may provide another incentive for the use of raw sequencing data.

## CONCLUSIONS AND FUTURE WORK

We have developed geno2pheno[ngs-freq], a free and publicly accessible web server for the rapid genotypic interpretation of viral drug resistance in NGS samples. geno2pheno[ngs-freq] is the first service that enables the application of geno2pheno[resistance] on NGS samples and, to the best of our knowledge, provides the first interpretation engine for NGS samples from HCV. We developed a new visualization of drug resistance (Figure 2) that enhances the interpretability of both algorithm-driven and rules-based interpretation engines. Due to its reliance on frequency files, geno2pheno[ngs-freq] can be integrated into existing NGS pipelines for interpreting viral resistance. By providing a means of exploring drug resistance for viral populations at multiple prevalence levels, we expect that geno2pheno[ngs-freq] will contribute to making clinical decisions and researching the impact of low-prevalence resistance mutations.

In future versions of geno2pheno[ngs-freq], we plan to improve the interpretability of the service, for example by annotating called variants using data from the literature. We also intend to incorporate further prediction models, particularly for identifying the coreceptor that is used by HIV (46,47) and for predicting the susceptibility of HIV-1 towards integrase strand transfer inhibitors. At a later point in time, support for samples from other viral species such as HBV, for which the emergence of resistance is relevant (48,49), could be added. Last, we are working towards providing an application programming interface that will be made available for the use in research settings.

## DATA AVAILABILITY

geno2pheno[ngs-freq] (http://ngs.geno2pheno.org) identifies viral resistance based on the approaches of geno2pheno[resistance] (www.geno2pheno.org) and geno2pheno[hcv] (http://hcv.geno2pheno.org). geno2pheno[ngs-freq] relies on geno2pheno[mutext] (http://align.geno2pheno.org) for performing local pairwise alignments.

## Supplementary Material

Supplementary Data
