# Supplementary material for: geno2pheno[ngs-freq]: a genotypic interpretation system for identifying viral drug resistance using next-generation sequencing data
Source: Nucleic Acids Res. 2018 May 1;46(Web Server issue):W271–7. doi: 10.1093/nar/gky349 (PMC6031006; doi:10.1093/nar/gky349)
Supplement: Supplementary Data [file gky349_supplemental_files.zip › g2p_ngs_supplement.pdf]

Supplementary File S1. Codon frequency file for the HIV-1 case study.

Supplementary File S2. Single-nucleotide frequency file for the HCV case study.

Supplementary Text S3. Generation of consensus sequences from single-nucleotide frequency files.

For each prevalence cutoff $c_F \in [0,1]$ provided for a single-nucleotide frequency file $F \in \mathbb{N}_0^{m \times n}$, the web service generates a consensus sequence in the following manner. Let $d_i = \sum_{j=1}^{n} f_{i,j}$ indicate the depth of coverage at position $i \in \{1, \dots, m\}$ over all considered nucleotides $j \in \mathcal{A}$. For the identification of codon abundances, only positions i that represent the first position of a coding triplet consisting of positions i, $i+1$, and $i+2$ are considered. For each such value of i, all possible combinations of observed nucleotides at the positions $i, i+1$, and $i+2$ are taken into account. Without loss of generality, assume that nucleotides $\in \mathcal{A}$, $k \in \mathcal{A}$, and $l \in \mathcal{A}$ are observed at the three respective positions. Then, the following computations are performed. Under the assumption of positional independence, the single –nucleotide prevalence ratios $x_{i,j} = \frac{f_{i,j}}{d_i}$, $x_{i+1,k} = \frac{f_{i+1,k}}{d_{i+1}}$,and $x_{i+2,l} = \frac{f_{i+2,l}}{d_{i+2}}$ are computed to determine the prevalence ratio $x_{i,j \times k \times l} = x_{i,j}\, x_{i+1,k}\, x_{i+2,l}$ at which the codon $j \times k \times l$ is expected to be observed. If $D_i = \min_x \{d_x | x \in \{i, i+1, i+2\}$ indicates the maximally possible codon coverage, the expected number of read supporting codon $j \times k \times l$ is given by $x_{j \times k \times l}\, D_i$.

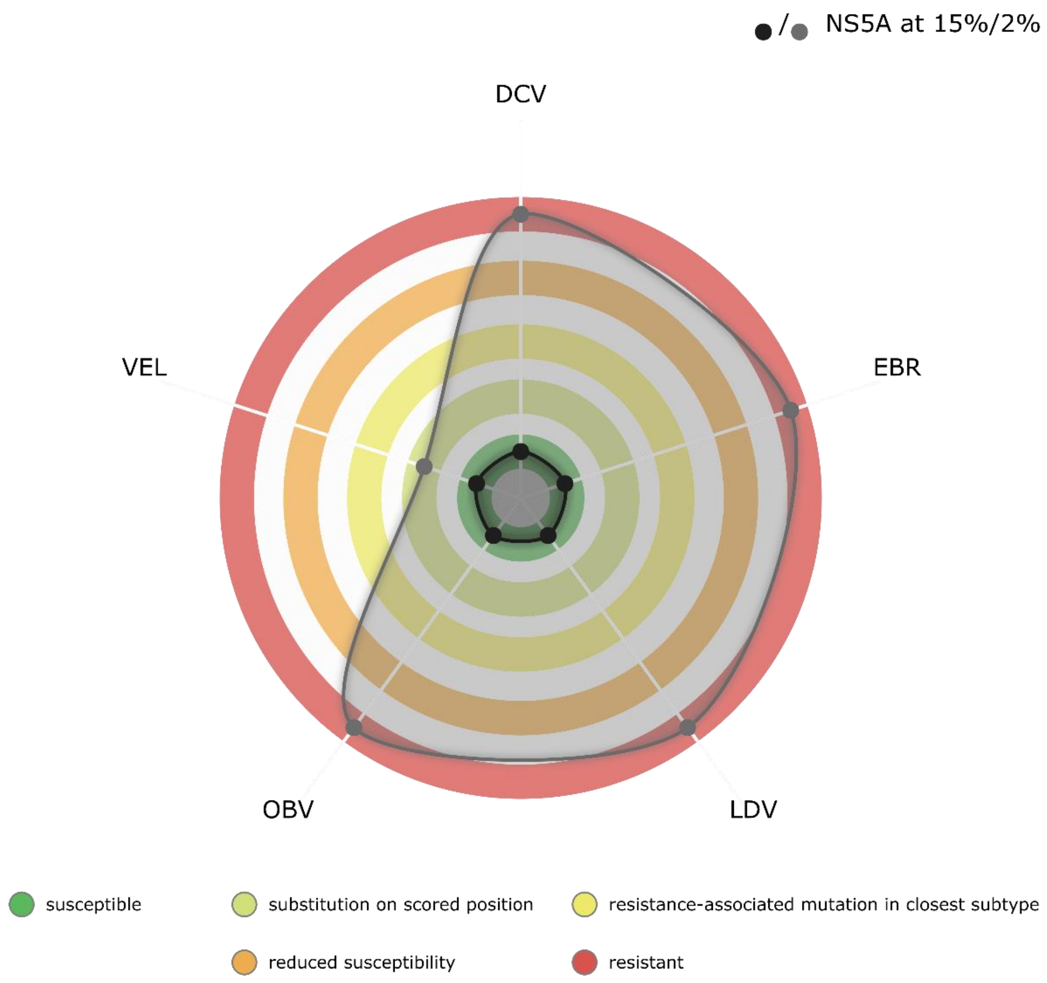

Supplementary Figure S4. Radar plot of predicted viral drug resistance for the HCV sample described in the second case study. Each spoke of the plot relates to an antiviral drug and the colored circle sectors indicate different levels of drug resistance. Predicted drug resistance is indicated by two surfaces: one surface showing the drug resistance estimate for the consensus sequence based on the personal prevalence cutoff at 2% and the other for the reference prevalence cutoff at 15%. The points defining the surfaces are based on the level of resistance that the set of rules assigns to individual amino acids in the sequence.
